# When Good Kinases Go Rogue: GSK3, p38 MAPK and CDKs as Therapeutic Targets for Alzheimer’s and Huntington’s Disease

**DOI:** 10.3390/ijms22115911

**Published:** 2021-05-31

**Authors:** Santosh R. D’Mello

**Affiliations:** Neugeneron, Dallas, TX 75243, USA; dmello@neugeneron.com; Tel.: +1-214-342-8105

**Keywords:** neurodegenerative diseases, cell cycle, Tau, Aβ, huntingtin, neuroinflammation, drug discovery

## Abstract

Alzheimer’s disease (AD) is a mostly sporadic brain disorder characterized by cognitive decline resulting from selective neurodegeneration in the hippocampus and cerebral cortex whereas Huntington’s disease (HD) is a monogenic inherited disorder characterized by motor abnormalities and psychiatric disturbances resulting from selective neurodegeneration in the striatum. Although there have been numerous clinical trials for these diseases, they have been unsuccessful. Research conducted over the past three decades by a large number of laboratories has demonstrated that abnormal actions of common kinases play a key role in the pathogenesis of both AD and HD as well as several other neurodegenerative diseases. Prominent among these kinases are glycogen synthase kinase (GSK3), p38 mitogen-activated protein kinase (MAPK) and some of the cyclin-dependent kinases (CDKs). After a brief summary of the molecular and cell biology of AD and HD this review covers what is known about the role of these three groups of kinases in the brain and in the pathogenesis of the two neurodegenerative disorders. The potential of targeting GSK3, p38 MAPK and CDKS as effective therapeutics is also discussed as is a brief discussion on the utilization of recently developed drugs that simultaneously target two or all three of these groups of kinases. Multi-kinase inhibitors either by themselves or in combination with strategies currently being used such as immunotherapy or secretase inhibitors for AD and knockdown for HD could represent a more effective therapeutic approach for these fatal neurodegenerative diseases.

## 1. Introduction

Alzheimer’s disease (AD) is a mostly sporadic brain disorder characterized by progressive cognitive decline resulting from neurodegeneration that starts in the entorhinal cortex and progresses to the hippocampus and large portions of the cerebral cortex [1,2,3,4]. In contrast, Huntington’s disease is a monogenic inherited disorder characterized by progressive motor deficits and psychiatric disturbances resulting from neurodegeneration largely localized to the striatum and to a lesser extent specific neuronal populations in the cerebral cortex [5,6,7]. It is generally believed that neurodegeneration in AD is caused by elevated levels of an abnormal form of the amyloid-β (Aβ) peptide, Aβ42, that forms extracellular oligomers and aggregates, and the hyper-phosphorylation of the microtubule-associated protein Tau, promoting its disassociation from axonal microtubules and deposition in insoluble neurofibrillary fibrillary tangles [1,2,3,4]. Oligomeric forms of both Aβ42 and Tau affect synaptic function and neuronal survival. Additionally, glial cells also become dysfunctional contributing to disease pathogenesis. HD is caused by the abnormal expansion of a polyglutamine (polyQ) stretch within the N-terminal region of the huntingtin (Htt) protein resulting in its misfolding. Although how the production of mutant polyQ-expanded Htt (or mut-Htt) causes neurodegeneration is unresolved, the consensus view is that the mut-Htt protein is abnormally phosphorylated, proteolytically cleaved and aggregates of the N-terminus fragment of the cleaved protein accumulate in the nucleus disrupting transcription. Aggregates of mut-Htt are also found in the cytoplasm where they are disrupt a variety of cellular processes [5,6,7]. Although with different etiologies (largely sporadic versus strictly genetic) and affecting largely non-overlapping neuronal populations resulting in distinct clinical features, the two diseases (and many other age-associated neurodegenerative diseases) share a variety of molecular and cellular commonalities. In addition to abnormal protein aggregation, these include mitochondrial dysfunction, elevated oxidative stress, endoplasmic reticulum (ER) stress, deregulation of autophagy, and abnormal post-translational modifications resulting from deregulated activity and functioning of the enzymes that mediate these modifications that in turn impact the functioning of other enzymes, macromolecules and cellular processes not just in neurons, but glial cells also. Additionally, aberrant activation of astrocytes and microglia induce the release of toxic cytokines that injure neurons. Among the enzymes that have been most convincingly implicated in the pathogenesis of neurodegenerative diseases are protein kinases, a family of about 500 proteins, most of which fall into two types-serine/threonine kinases, which phosphorylate serines and/or threonines in their target protein, and the tyrosine kinases. Among the hundreds of serine-threonine protein kinases, only a small number have been seriously implicated in the pathogenesis of neurodegenerative diseases and within this group, less than a dozen common kinases have been described to play a key role in diverse neurodegenerative disease with distinct etiologies. Prominent among these are glycogen synthase kinases (GSKs), the mitogen-activated protein kinases (MAPKs) and the cyclin-dependent kinases (CDKs). This review focuses on these three kinases and their contributions to the pathogenesis of AD and HD. The review starts with a brief overview of AD and HD, focusing on the cellular and molecular abnormalities. A summary of the properties of each of the three kinases is then described before reviewing evidence for their involvement in each of the two neurodegenerative disease, and their merits as targets for the development of therapeutics for the diseases. Although much of the currently-developed therapeutic approaches for AD target Aβ or Tau [8,9] and mut-Htt for HD [10], this review aims to impress upon the reader that the neuropathological action of these proteins requires or is regulated by the three kinases that the review focuses on and makes the case for targeting these kinases, possibly in combination with approaches that are currently being clinically tested, such as genetic knockdown or immunotherapy.

## 2. The Diseases

### 2.1. Alzheimer’s Disease

AD is a predominantly sporadic disorder with several risk factors the best defined of which are aging as well as mutations and variations of a large number of susceptibility genes, among which the most studied and accepted are expression of the ApoE4 (apolipoprotein E4) isoform and mutations in TREM2 (triggering receptor expressed on myeloid cells-2), a protein expressed within the brain by microglia [1,2,3,4]. For a comprehensive listing and description of the susceptibility genes for AD the reader is referred to a review by Verheijen and Sleegers [11]. In ~4% of the total cases, however, AD is familial, caused by mutations in genes including those encoding Aβ precursor protein (APP), presenilin-1 (PS1) and presenilin-2 (PS2) [1,2]. APP is the protein from which Aβ peptide is derived from by proteolysis while PS1 or PS2 are the catalytic components of the γ-secretase complex, which along with b-secretase/BACE1, cleaves APP to produce Aβ. Altered activities of BACE1 and γ-secretase in AD results in increased production of Aβ. This along with reduced proteolytic degradation of Aβ extracellularly by proteases, such as insulin-degrading enzyme (IDE) and neprilysin, results in its accumulation as neurotoxic Aβ oligomers and fibrils [12,13]. Amyloid plaques, a neuropathological hallmark of AD composed of insoluble deposits of Aβ aggregates are widely believed to represent sites at which oligomeric Aβ species are sequestered as a protective mechanism against their toxicity [14]. Hyperphosphorylation of Tau results in its disassociation from axonal microtubules leading to destabilization of microtubules, thus affecting axonal structure and transport [14,15]. Disassociated Tau forms neurotoxic fibrils and insoluble fibrillary tangles (NFTs) in the cytoplasm of neurons representing another neuropathological hall mark of AD [15]. Although the three AD-causing genetic mutations are linked to Aβ synthesis, NFTs are more closely correlated with cognitive impairments [15,16]. Furthermore, although neuronal death is the defining feature of the disease, it is synaptic degeneration and dysfunction that initiates cognitive impairment the clinical feature that best characterizes AD [17,18,19,20]. The accumulation of extracellular Aβ plaques triggers the activation of microglia and astrocytes that release inflammatory cytokines. Although with the purpose of eliminating the plaques, chronic release of inflammatory cytokines, a process referred to as neuroinflammation, promotes synapse elimination and neuronal death [21,22]. The AD susceptibility genes, ApoE and TREM2, respectively, contribute to the clearance of Aβ and reducing neuroinflammation, respectively, providing an explanation for why variant and mutant isoforms deficient in these functions increases the risk for AD [23,24,25]. Dysfunction of astrocytes and microglia also contribute to deregulation of glutamate homeostasis, resulting in the elevation of extracellular glutamate and the triggering of excitotoxicity in neurons through the overactivation of NMDA receptors, a subtype of ionotropic glutamate receptors [26]. Since astrocytes contribute to Aβ catabolism, astrocyte dysfunction could contribute to the pathogenic accumulation of Aβ [26]. Additionally, while astrocytes and microglia ultimately cause neuroinflammation, under normal circumstances these cells secrete cytokines that support the survival and functioning of neurons, a function which is lost when these cell types become functionally compromised [26,27]. Although less studied than astrocytes and microglia, emerging evidence suggests that impaired functioning of oligodendrocytes and their progenitor cells (which remain through adulthood) may also play a causal role in AD. For example, breakdown of myelin is enhanced in AD, occurs early in the diseases process and has been found to correlate with the spreading of NFTs [28,29,30,31], and both oligodendrocytes and oligodendrocyte progenitor cells (OPCs) phagocytose Aβ a function that is impacted in AD [32]. One recent study described that oligodendrocytes may facilitate the spread of pathogenic Tau in the brain even in the absence of neuronal Tau [33]. Taken together, although the overwhelming focus on Aβ and Tau pathology has led to a neuron-centric model for AD pathogenesis, findings from more recent research is making it increasingly clear that dysfunction of glial cell types is also an important contributor.

Another widely described pathogenic event in AD is the aberrant entry of neurons into the cell cycle leading to mitotic failure and death [34,35,36,37,38,39]. Although the significance of aberrant cell cycle entry to the triggering of AD is still to be fully resolved, it is considered by many to be, along with Aβ and Tau pathology, a third major cellular abnormality underlying neurodegeneration in AD (and several other neurodegenerative diseases). Several lines of evidence support this conclusion, including the activation of cell cycle promoting cyclin-dependent kinases (CDKs) and cyclins, misregulation of other cell cycle regulatory proteins, including retinoblastoma protein (Rb), CDC25 phosphatases, and the increase in aneuploidy and mitotic morphology in degenerating neurons and areas of the brains of AD mice and patients [35,36,40,41,42,43]. It has been suggested that aneuploidic neurons can survive in the brain for many years but selectively die during aging providing an explanation for the late-onset neurodegeneration [34,44]. Work in mice has revealed that increase in cell cycle markers precedes the elevation in Aβ and Tau hyperphosphorylation [45]. Consistent with a causal role for cell cycle entry, CDK inhibitors block neuronal death in cell culture models of AD (Rao et al., 2020; Herrup 2012).

Although the precise mechanisms and extents of contribution remain unclear, it is widely accepted that oligomeric Aβ and hyperphosphorylated Tau fibrils along with excitotoxicity, neuroinflammation and abortive cell cycle entry and a variety of other cellular disturbances, including impaired cholinergic signaling [46], ER stress [47,48], disrupted autophagic clearance of protein aggregates [49,50,51,52,53], mitochondrial dysfunction [54], oxidative stress [55], and deregulation of iron metabolism [56] combine to cause progressive synaptic failure and neuronal death in the AD brain.

There are no effective disease-modifying drugs for AD with existing medications, primarily acetylcholine esterase inhibitors and the NMDA receptor antagonist, memantine, serving to modestly relieve symptoms [57]. Clinical trials conducted so far aimed at developing disease-modifying therapies have all been unsuccessful. Recent studies have concluded that molecular changes in the AD brain begin decades before neuropathology and behavioral deficits are displayed by patients indicating that therapeutic intervention must be delivered early in the disease process for effectiveness [58,59]. In this regard it is noteworthy that much of the recent efforts at developing disease-modifying therapies for AD have focused on Aβ and Tau and include immunotherapy, drugs to reduce Aβ production or increase clearance, and drugs to inhibit Tau phosphorylation or polymerization, but these have yet to show success in the clinic [57,60,61]. Given the evidence that abnormal activation of kinases, such as GSK3, p38 MAP kinase and CDKs is an early and critical event in the disease process [62,63] that precedes Aβ and Tau oligomerization/fibrillization and actually contribute to it, stronger emphasis must be placed on the development of kinase inhibitors that may be utilized along with Aβ and Tau-targeted therapies.

### 2.2. Huntington’s Disease (HD)

HD is an autosomal dominant, progressive and fatal neurodegenerative disease caused by the abnormal expansion of a CAG trinucleotide repeat located in exon 1 of the Htt gene resulting in the expansion of a polyglutamine stretch in the N-terminus region of the Htt protein, rendering the mutant protein prone to misfolding [5,6,7]. Wild-type Htt is a large protein (~350 kD) present in both the cytoplasm and nucleus where it interacts with a large number of proteins [64]. Htt plays a critical role during embryogenesis and, consistent with the large repertoire of interacting-proteins, is involved in numerous cellular processes postnatally including regulation of transcription [65], nucleocytoplasmic trafficking [66], axonal transport [67], DNA repair [68], autophagy [69], mitophagy [70] and cell division [71]. Although mut-Htt (like wild-type Htt) is expressed ubiquitously, HD is characterized by selective degeneration of medium-spiny neurons (MSNs) of the striatum and, to a lesser extent, pyramidal neurons in specific layers of the cortex [5]. Mut-Htt forms oligomers and large aggregates in the nucleus and cytoplasm that disrupt neuronal function and promote degeneration in vulnerable brain regions. Although not well studied, neuronal loss and pathology has also been described in the hippocampus, thalamus and cerebellum in HD [72]. Convincing evidence indicates that mut-Htt oligomers are the toxic species whereas the aggregates that form inclusions may be protective [73,74,75]. It is generally recognized that both loss of normal function and acquisition of a toxic function by the mutant protein are involved in HD pathogenesis although the underlying molecular mechanisms remain largely unresolved. Dysregulation in neurotransmitter systems, including the glutaminergic, GABAergic and dopaminergic systems have been documented in HD mouse models and patients [76,77]. A role for mitochondrial dysfunction and oxidative stress has been amply documented [5,78,79]. A major cause of synaptic and neuronal loss in HD is excitotoxicity resulting from glutamate dyshomeostasis [77]. Chronic activation of microglia and astrocytes and the presence of neuroinflammation is a consistent feature of HD [80,81,82]. It is known that mut-Htt promotes release of inflammatory cytokines from astrocytes and microglia [83,84]. As observed in AD and several other neurodegenerative diseases, abnormal immune system activation has been documented in HD patients and mice leading to the elevation of IL-6 and other inflammatory cytokines and this has been reported to occur even before the appearance of diseases symptoms suggesting that immune system dysfunction contributes to brain pathology [84,85]. Interestingly, the cytokines released in HD are fewer and more localized within the striatum and other brain regions than the more general pattern seen in AD and other neurodegenerative diseases [85]. Whether neuroinflammation triggers development of neuropathology and behavioral deficits in HD has yet to be firmly established, there is wide consensus that neuronal death during the diseases provokes an neuroinflammatory response that exacerbates neurodegeneration [5,82].

While clearly a consequence of polyQ-expansion, other regions within the Htt protein and post-translational modifications within them are critically involved in regulating disease pathogenesis. Most studied in this regard is the N-terminus region of the protein containing the polyQ expansion. The highly-conserved 17 amino acids before the polyQ stretch, referred to as N17, which targets Htt to different subcellular compartments and regulates its folding, protein-protein interactions and clearance, is phosphorylated at three residues Thr3, Ser13 and Ser16 [86,87,88]. Phosphorylation at Ser13 and Ser16 is reduced in polyQ-expanded Htt whereas replacement of these two residues with phosphomimetic residues inhibits aggregation and neurotoxicity by mut-Htt both in tissue culture and in mice [86,87]. Initial studies pointed to IKKβ as being the kinase that phosphorylates Ser13 and Ser17 [86] although a subsequent study reported that the phosphorylating kinase is Casein Kinase-2 (CK2) [89]. Similarly, phosphorylation of Thr3 by IKK reduces aggregation and neurotoxicity of mut-Htt in cultured cells and in *Drosophila* [88,90]. Additionally, deregulations in signaling pathways cause disease-associated posttranslational modifications in other proteins within neurons, including DARP32 (dopamine and cAMP-regulated phosphoprotein), Tau, the CREB transcription factor, the mitochondria fission regulating protein Drp1 (dynamin-related protein 1), and several others [91]. The deregulations in many signaling pathways occur prior to mut-Htt aggregation and some evidence indicates that they play a key role in promoting mut-Htt aggregation and disease pathogenesis [91]. 

An accumulating body of evidence supports a role for Tau dysregulation in HD disease pathogenesis. Tau phosphorylation is increased in vulnerable regions of the brains of HD mice and patients where it accumulates in aggregates [92,93,94,95,96]. Additionally, and as a result of splicing alterations, the expression levels of specific Tau isoforms are changed in HD brain tissue causing disruption of the nuclear membrane [97,98]. Based on the documented Tau pathology in HD several investigators have referred to HD as a tauopathy, placing it in the same disease category as AD [99,100,101,102]. 

Although a monogenic disorder caused by a single mutation (trinucleotide expansion) in a gene identified about 30 years ago and the availability of a variety of invertebrate and rodent models, HD remains without disease-modifying therapies. Clinical trials that have targeted intracellular alterations such as mitochondrial dysfunction, reactive oxygen species (ROS) accumulation and oxidative damage, neurotransmitter systems, and pathways that modify mut-Htt modifications and aggregation has so far been ineffective. Some studies have described neuroprotection by histone deacetylase-3 (HDAC3)-selective inhibitors, both in cell culture and mouse models of HD [103,104,105,106,107]. Based on the finding that knockdown of Htt in adult HD mice has no obvious adverse effects, much of the current therapeutic focus has been on approaches that lower Htt/mut-Htt expression [108,109]. These approaches include ones targeting expression at the mRNA level, including antisense oligonucleotides (ASOs) and RNA interference or at the DNA level, including CRISPR-Cas9, transcription activator-like effector nuclease (TALEN) and zing-finger proteins (ZNFs) [108,109]. Based on bioinformatic analyses, allele-selective ASOs have recently been developed that knockdown mut-Htt allele selectively. Although a promising avenue, the issue of off-target effects of the ASOs and other Htt-knockdown approaches in humans is a concern. A recent report described the identification of a chemical that binds expanded CAG tracts and promotes contraction of the repeats, which could have value in the treatment of HD and other CAG-repeat expansion disorders [110,111].

## 3. The Enzymes

### 3.1. (A) GSK3

GSKs, a family of three serine-threonine protein kinases—GSK1, GSK2 and GSK3—were identified by their ability to phosphorylate glycogen synthase, the rate limiting enzyme of glycogen metabolism [112,113,114]. GSK3 is the best studied of the GSKs, which is known to regulate a variety of cellular functions besides glucose metabolism. As in much of the literature, for the rest of this review GSK refers to the GSK3 protein. Two paralogs, commonly referred to as isoforms, of GSK3 are produced from separate genes: GSK-3α and GSK3β with molecular weights of 51 kDa and 47 kDa, respectively [115]. The two isoforms share ~95% amino acid identity and are therefore thought to phosphorylate many of the same proteins. Although involved in common functions, GSK3α and GSK3β have non-overlapping functions best exemplified by the finding that knockout of GSK3β in mice generally results in embryonic lethality whereas global knockout of GSK3α results in viable animals although these mice develop age-related pathologies and a slightly shortened lifespan [116,117,118]. Specific non-overlapping functions for the two GSK isoforms in the brain have been described [119,120,121,122]. GSK3 is unusual among protein kinases in that it is generally constitutively-active making its inhibition, rather than activation, the primary mode of its regulation [123]. Alterations in GSK3 activity depends on the extent of phosphorylation of Ser-9 in GSK3β (or Ser-21 in GSK3α) which inhibits constitutive activity, and the phosphorylation at Tyr216 of GSK3β (Tyr279 of GSK3α) which restores and increases enzyme activity. Phosphorylation at other residues also contributes to increased GSK3 activity. In addition, GSK3 activity and function depend on its subcellular localization, the subcellular localization of its substrates, the level of its substrates, and its inclusion in multi-protein complexes that can control access to its substrates [123]. It has been reported that the activity of GSK3 can be increased through cleavage by calpains [124,125,126,127] and matrix metalloproteinase-2 [128] and inhibited by mono-ADP-ribosylation [129,130], acetylation [131,132] and citrullination [133]. Although largely a cytosolic protein, GSK3 is present in the nucleus and mitochondria. In the nucleus, phosphorylation by GSK3 modulates the activities and functions of many transcriptional regulators, many of which are known to promote neurodegeneration, including c-jun [134,135], HDAC3 (histone deacetylase-3) [136], HDAC4 [137] and DNMT1 (DNA methyltransferase-1) [138]. In contrast to most other kinases, the substrates of GSK3 generally (but not always) need to phosphorylated by another kinase before they are phosphorylated by GSK3 [123]. Despite this requirement, GSK3 is believed to phosphorylate more substrates than any other protein kinase with over 100 substrates being identified in biochemical studies and a much larger number based on computational analyses [139,140].

#### 3.1.1. GSK3 in the Brain

GSK3α and GSK3β are differently-regulated during brain development with GSK3β being highly expressed during neurogenesis while GSK3α is poorly expressed during that period. In the mature brain GSK3β is widely present whereas GSK3α shows a more restricted pattern of expression with highest expression in in the cerebral cortex, striatum hippocampus, and Purkinje cells of the cerebellum [141]. Likely because of it abundance and widespread expression in the brain and the prenatal lethality of GSK-3α knockout mice, research on the role of GSK3 in the brain have until recently focused almost exclusively on GSK3β. Multiple roles have been proposed for GSK3β in the adult brain including the negative regulation of neurogenesis in the hippocampus [142,143,144], regulation of synaptic plasticity, learning and memory [145,146,147,148], and stimulation of the inflammatory function of microglia [149,150,151]. While with multiple beneficial functions, elevated GSK3 activity promotes death of neurons in culture [152,153,154] and in the brains of mice [155].

#### 3.1.2. Roles of GSK3 in AD

**Figure 1:**
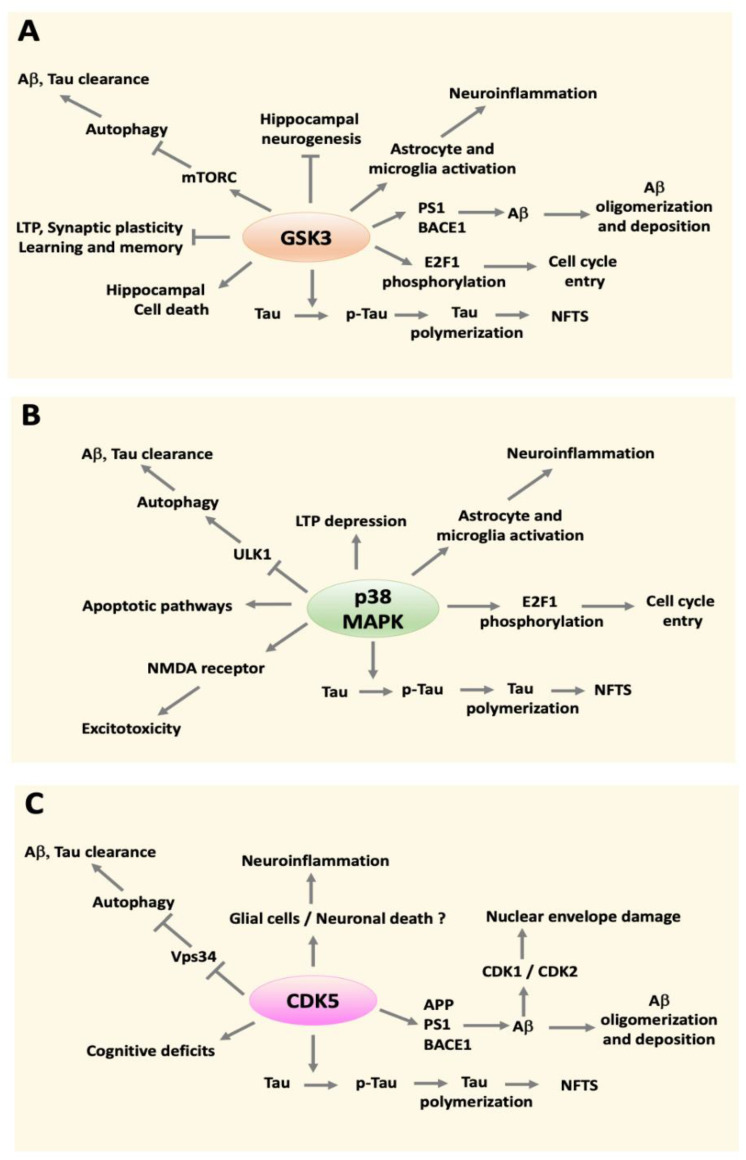
Effects of elevated GSK3, p38 MAPK and CDK5 activity in models of AD. (**A**) GSK3 increases Aβ levels by activating PS1 [165,166,167] and increasing the expression of BACE1 [168,169], impairs Aβ and Tau clearance by inhibiting autophagy [172,173], promotes neuroinflammation [147,149,151,174], depresses LTP [175], and negatively regulates synaptic plasticity and learning/memory [176,177]. Through E2F1 phosphorylation, GSK3 promotes abortive cell cycle entry [178,179] and by phosphorylation Tau causes its disassociation from microtubules and assembly into fibrils and NFTs [15,158,159,160,161]. GSK3 also inhibits adult neurogenesis and promotes neuronal death in the hippocampus [143]. Although inhibiting GSK3 protects in experimental models of HD, less is known about the mechanism by which elevated GSK3 activity promotes neuronal death in HD. Several lines of evidence indicate that GSK3 activates HDAC3-mediated neurotoxicity through its phosphorylation [106]. GSK3 also activates caspase-3, phosphorylates Tau, promotes neuroinflammation and causes cognitive impairment in HD models [180]. (**B**) In astrocytes and microglia p38 MAP promotes release of inflammatory cytokines resulting in chronic neuroinflammation and consequently to neurodegeneration [181,182,183]. Within neurons p38 has been shown to have several effects that promote neurodegeneration including the activation of apoptotic signaling pathways [182,184,185,186], the phosphorylation of Tau [187,188,189], the phosphorylation of E2F1 [190,191], inhibition of LTP [192,193,194], the promotion of excitotoxicity through activation of the NMDA glutamate receptor [195,196,197], and the inhibition of autophagy through the phosphorylation of the ULK1 complex [198,199,200], which impairs the ability of neurons and glial cells to clear Tau and Aβ aggregates. It should be noted that the actions depicted in the figure pertain to p38α, p38βand p38δ MAPK, and most specifically to p38α. In contrast to the other p38 MAPKs, p38γ is believed to have neuroprotective actions in the context of AD. Although inhibiting p38 MAPK promotes neurodegeneration in HD by acting both in neurons and in glial cells, the mechanisms involved are unclear. Most emphasis has been placed on the mechanisms by which p38 MAPK activity is increased in HD. (**C**) Not shown in the figure are the actions of cell cycle-promoting CDKs, which are aberrantly activated in neurons in the AD brain resulting in an abortive entry into the cell cycle culminating in cell death [34,35,36,201,202,203]. Cell cycle promoting CDKs also promote neurodegeneration by other mechanisms, such as increasing APP processing and inhibiting autophagy [204]. Of the CDKs, CDK5 is most involved on AD. By stimulating PS1 and BACE1 activity, CDK5 increases Aβ levels by both transcriptional mechanisms and through PS1 and BACE1 activation [205,206,207]. Elevated Aβ, through activation of CDK1, 2 and 4, leads to the phosphorylation of lamins and nuclear membrane damage [208]. CDK5 is also a major Tau kinase and causes Tau dysfunction [209]. Through phosphorylation of Vps35, CDK inhibits autophagic clearance of Aβ and Tau [204,210,211]. CDK5 produces neuroinflammation both by promoting neuronal death and more directly by stimulating lysophosphatidylcholine release from glia [212]. CDK5 also plays a key role in HD pathogenesis by inducing mitochondrial fission [213], generating oxidative stress and promoting excitotoxicity [214,215], by the aberrant phosphorylation of DARPP32 [216], by increasing expression of the pro-apoptotic protein, c-jun [217], and increasing activity of specific cell cycle-promoting CDKs through the stimulation of expression of their cognate cyclins [218].

GSK activity is upregulated in the hippocampus of AD patients and is associated with phosphorylated Tau and NFTs [156,157]. Several studies have established that Tau phosphorylates GSK3 at multiple disease-relevant sites in mouse models of AD and in the patient brains, and is a central kinase in Tau hyperphosphorylation causing disassociation of Tau from microtubules and fibrillization [15,158,159,160,161] (Figure 1A). GSK3 has also been reported to stimulate Aβ production whereas its pharmacological inhibition or knockdown reduces processing of APP to Aβ [161,162,163,164] (Figure 1A). Consistently, GSK3 over-activation can increase the activity of PS1 [165,166,167] and the expression of BACE1 [168,169]. Elevated Aβ activates GSK, which then through increased processing of APP also produces more Aβ resulting in a positive-feedback loop. Aβ-mediated GSK3 activation also increases Tau phosphorylation and induces its fibrillization linking Aβ to Tau dysfunction via GSK3 [170,171]. Inhibition of GSK3 reduces Aβ levels, tau hyperphosphorylation, and cognitive deficits in mice [15,158] (Figure 1A). 

As described above, an important cellular mechanism that is impaired in AD is autophagy, a process that degrades and clears misfolded and aggregated proteins as well as other dysfunctional macromolecules and organelles. In the context of AD, impaired autophagy is thought to be responsible in major part for the accumulation of Aβ and Tau aggregates [50,52]. Not surprising, drugs that activate autophagy have been found to be neuroprotective in cell culture and mouse models of AD [172,219,220,221]. GSK3 is a well-established inhibitor of autophagy and some studies have shown that inhibitors of GSK3 reduce AD pathology by stimulating autophagy [172,173].

In addition to directly promoting neuronal death in neurons, GSK3 promotes neuroinflammation in AD though the regulation of the expression and release of harmful cytokines from glial cells [147,149,151,174]. Adult neurogenesis, which serve to replace neurons, is sharply reduced in the AD hippocampus exacerbating the loss of hippocampal function [143]. This reduction of neurogenesis in AD has been found to be GSK3-dependent. Inhibiting GSK3 promotes neurogenesis and reduces neuronal death in the adult hippocampus [142]. In sum, convincing results indicate that GSK3 activation occurs early in the disease process and promotes all the major pathogenic changes that cause or contribute to AD pathogenesis [62,222,223,224] (Figure 1A).

#### 3.1.3. GSK3 as a Therapeutic Target in AD

Several brain-penetrant GSK3 inhibitors have been evaluated in cell culture, slice and animal models of AD. A number of laboratories have tested lithium, a drug commonly used in the treatment of bipolar mood depression but known to inhibit GSK3 [225,226]. Lithium administration inhibits GSK3 in mouse models of AD and promotes Aβ and Tau neuropathology, facilitates LTP (long-term potentiation) induction and improves cognitive performance [175,227,228,229]. Lithium also reduces neuronal dysfunction in an *Drosophila* model of AD in which Aβ is inducibly-overexpressed in adult flies [230]. However, lithium is a poor drug candidate for use in humans because of its narrow therapeutic window and serious side effects, including neurotoxicity, particularly in the elderly [231,232,233]. Lithium is also known to inhibit other enzymes and that its beneficial effects in AD mice is due to inhibition of GSK3 has yet to be established [234,235]. Despite its limitations, lithium has been tested in patients with mild cognitive impairment (MCI) and AD with confusing results. While a couple of studies found slight improvement in cognitive function [236,237], others studies reported no improvement or even an increase in dementia [238,239,240]. Tideglusib (also referred to as NP12 or NP031112) is another selective GSK3 inhibitor that reduces Tau phosphorylation, decreases Aβ deposition, inhibits plaque-associated gliosis, protects neurons in the entorhinal cortex and hippocampus against cell death, and reduces memory deficits in AD mice [176]. Although tideglusib was well-tolerated in AD patients, it displayed no clinical benefit in an initial safety study and a Phase-II trial [241,242]. A GSK3 inhibitor developed by AstraZeneca, AZD1080, reduces Tau hyperphosphorylation in rats and synaptic plasticity deficits in rodents but was abandoned in Phase II trials because of severe side effects [243,244]. Given the impressive effects of GSK3 inhibitors in a variety of in cell culture and preclinical studies, the lack of efficacy in AD patients is surprising. It is likely that treatment in patients requires initiation of treatment earlier in the disease process. Alternatively, it is possible that the complex mechanisms of AD pathogenesis in humans will require the simultaneous targeting of multiple molecules, including but not limited to GSK3. The strategy of combinatorial drug therapy is well-accepted in the treatment of cancer [245,246,247]. 

Another highly potent GSK3 inhibitor developed by AstraZeneca belonging to the pyrazine class and with excellent drug-properties, AZD2858, was effective at reducing Tau phosphorylation and gliosis in the hippocampus but did not proceed to clinical trials because of failure to pass preclinical toxicology studies [243]. A recent study described that a GSK inhibitor developed by Sanofi, SAR502250, reduced Tau hyperphosphorylation in the cortex and spinal cord in P301L human transgenic mice [248]. Additionally, this compound protected against neuronal loss resulting from Aβ treatment and reduced cognitive impairment in two separate mouse models of AD, a transgenic mouse model and the Aβ-infusion model [248]. The effect of SAR502250 on Aβ or Tau neuropathology or on neuroinflammation was not examined in this study and therefore how it exerts its beneficial effects have yet to be determined. A thiazole GSK3 inhibitor, AR-A014418, inhibits tau phosphorylation and is neuroprotective in cell culture and inhibited Aβ neurotoxicity in hippocampal slices [249]. AR-A014418 also reverses axonal transport defects and behavioral deficits in Tau-overexpressing *Drosophila* [250]. However, another study conducted in young rats reported that AR-A014418 did not inhibit Tau phosphorylation [251]. Chronic low-dose administration of another GSK3 inhibitor, AM404, reduced Aβ production, tau hyperphosphorylation, neuroinflammation and cognitive impairment in AD mice [244,252]. However, at higher doses this compound had detrimental effects on brain function [253]. 

Among other GSK3 inhibitors that have been tested to a limited extent in AD-related models are a set of isonicotinamides, which reduce Tau activity in triple-transgenic AD mice [254]. In another study several GSK3 inhibitors belonging to the aminopyrimidine, indurubin, alsterpaullone, thiazole classes were tested for their ability to suppress phosphorylation of Tau at Ser-396 in normal rats, increased phosphorylation of which is associated with AD [251]. It was found that CHIR98014, an aminopyrimidine, reduced Tau phosphorylation in the cortex and hippocampus, while two other GSK3-inhibiting compounds, an alsterpaullone and SB216763, reduced phosphorylation only in the hippocampus suggesting region-specific regulatory mechanisms for Tau phosphorylation, including differences in the pattern of Tau phosphorylation or the involvement of another kinase or phosphatase in one brain region and not another. Another study described that SB216763, an ATP competitive-inhibitor of GSK3, protected against neuronal damage resulting from intracerebroventricular infusion of Aβ in mice, but had very modest effects on gliosis and behavioral deficits [255]. Surprisingly, in control mice SB216763 induced inflammation and behavioral deficits possibly due to inhibition of constitutive GSK3 activity [255]. Antisense oligonucleotides against GSK3β have also been tested in SAMP8 mice, which display accelerated aging along with increased Aβ levels, Tau hyperphosphorylation, neuroinflammation and cognitive deficits and have hence been used as a model of AD [177,256,257]. Knockdown of GSK3β reduced oxidative stress in the brain and improved learning and memory of SAMP8 mice [177].

#### 3.1.4. Roles of GSK3 in HD

Although predominantly cytosolic, GSK3 accumulates and co-localizes in lipid rafts with mut-Htt in cultured neurons and in presymptomatic HD mice, an alteration that has been suggested to contribute to disease development [258]. While the striatum and the cortex are the most affected brain regions in HD, neuronal loss and dysfunction in the hippocampus has also been described [259,260,261,262] An upregulation of GSK3β expression (mRNA and protein) and activity has been observed in the hippocampus of both HD mice and patients and in [180]. In HD mice the increase in GSK3β induces Tau phosphorylation and caspase-3 activation in the dentate gyrus leading to neuronal death [180]. Other studies have also described GSK3-mediated Tau phosphorylation and aggregation in HD, raising the possibility of common pathogenic mechanisms in HD and AD, at least with relation to Tau [92]. In addition to its actions in neurons, increased GSK3β expression and activity is observed in astrocytes within the hippocampus where it promotes release of inflammatory cytokines [180]. Although the mechanism of increased GSK3 activity has not been studied, it is possible that it is a consequence of reduced activity of Akt in the HD brain [263]. It is well-established that phosphorylation of GSK3 by Akt inhibits its activity *in vitro* and *in vivo* [264,265].

#### 3.1.5. GSK3 as a Therapeutic Target in HD

Pharmacological inhibitors of GSK3 or expression of a dominant-negative form of GSK3β are neuroprotective in cell culture models of HD [106,258,266]. An important target of GSK3 is HDAC3, a protein that is required for the neurotoxic effect of mut-Htt in cultured neurons [106,107] and promotes neurodegeneration and behavioral deficits in HD mice [103,104,105]. Inhibition of GSK3 using pharmacologically or through expression of a dominant-negative construct inhibits both HDAC3 and mut-Htt toxicity in cultured neurons [106]. *In vivo* protection by GSK3β was reported in a *C. Elegan* model of HD, although the inhibitor used in the study was lithium, which as described above, has other cellular targets [267]. Chronic treatment of HD mice with lithium produces variable effects with some mice displaying marked improvement in motor function whereas other mice do not [268]. Life span was not extended by lithium treatment in any of the mice. Another study described substantial benefit of lithium in HD mice only when it was co-administered with valproic acid, which itself has multiple targets, including the inhibition of HDACs [269,270,271]. A similar requirement was described in another study that reported additive protective effects of lithium and the mTOR inhibitor, rapamycin, in a *Drosophila* model of HD [272]. These results suggest that multiple signaling molecules and pathways may be involved in HD pathogenesis and full rescue would therefore require inhibiting multiple targets.

In contrast to the conclusions of the aforementioned studies, the results of a few other studies indicate that GSK3 plays a neuroprotective role in HD. One study described reduced GSK3 activity in the striatum of HD mice and patients [273]. This study showed that moderate overexpression of GSK3β in the striatum of HD mice attenuates brain atrophy, motor impairment and cognitive deficits [273]. One study described that elevation in glycogen synthase activity leading to increased glycogen synthesis results in enhanced autophagic flux which protects against mut-Htt toxicity in cell culture models [274]. Although this would suggest that an increase in GSK3 activity would be neuroprotective, whether this is the case was not examined in the study. Whether some of these differences with regard to toxicity versus neuroprotection are due to opposing actions of the two GSK3 isoforms and/or selectivity of antibodies used with regard to the two isoforms is unclear.

### 3.2. (B) p38 MAPK

Along with the two other families of mitogen-activated protein kinases (MAPKs), JNKS (c-jun N-terminal kinases) and ERKS (extracellular signal-regulated kinases), the p38 MAPKs are enzymes that regulate cellular responses to a wide range of extracellular signals [275,276]. p38 MAPKs were previously referred to as stress-activated protein kinases (SAPKs) because they are activated by various stimuli that are stressful or noxious to cells, including osmotic stress, oxidative stress, heat shock, genotoxic and DNA-damaging agents, and pathogen proteins [276,277]. In many cases p38 MAPK responds to such cellular stresses by mediating an inflammatory response [278]. Mammals express four p38 MAPKs that are all about 38 kDa is size and encoded by distinct genes: p38α (MAPK14), p38β (MAPK11) p38γ (MAPK12) and p38δ (MAPK13) [277,279]. Analyses of the primary sequences of the four isoforms reveals over 60% overall sequence homology and greater than 90% homology in the kinase domains. Most studies have focused on p38α and p38β. p38α is expressed ubiquitously and highly, whereas p38β is expressed at lower levels and in many tissues with highest expression in the brain. The two proteins share functions, including the mediation of inflammatory responses and regulation of cell proliferation, differentiation, survival and death [275,277]. However, global knockout of p38α in mice causes embryonic lethality whereas p38β knockout mice are phenotypically normal and fertile indicating unique functions of p38α that cannot be compensated for by the other isoforms [280,281]. In contrast to the widespread expression of p38α and p38β, p38γ and p38δ are expressed in a tissue-specific manner and have more specialized functions although what these are have not been fully identified. Knockout mice lacking either p38γ and p38δ or double-knockout mice lacking both isoforms have on obvious abnormalities [280,281].

p38 MAPKs are activated by phosphorylation within a Thr-Gly-Tyr motif in the activation loop by the dual-specificity MAPK kinases, MKK3 and MKK6 [277,282]. A large number of cytoplasmic and nuclear proteins have been identified as p38 MAPK substrates, including scaffold proteins, cytoskeletal proteins, signaling proteins, chaperones, and transcriptional factors and regulators. To activate nuclear substrates, activated p38 translocates to the nucleus by mechanisms that have not been fully resolved (p38 MAPKs lack nuclear import or nuclear export motifs). Following substrate phosphorylation p38 MAPKs are inactivated by dephosphorylation of the Thr-Gly-Tyr motif by dual-specificity phosphatases (DUSPs), also known as MAPK phosphatases (MKPs) located in the cytoplasm and nucleus. Dephosphorylation by MKPs in necessary for translocation of p38 MAPK out of the nucleus. Inactivation of p38 MAPK is also promoted by transcriptional feedback, reduced activity of upstream kinases and the termination of the activating stimuli [277,282,283]. In some cases p38 MAPKs can be activated through phosphorylation of non-canonical residues independent of MKK3 and MKK6 [284,285]. Despite the high level of structural similarity, the four 38 isoforms are differentially sensitive to pharmacological inhibitors. For example, while p38α and p38β are sensitive to pyridinyl imidazole inhibitors, p38γ and p38δ are insensitive to them [286].

#### 3.2.1. p38 MAPK in the Brain

Most studies on the function of p38 MAPK in the brain have focused on p38α and p38β, which are expressed in neurons as well as astrocytes, microglia and oligodendrocytes [283,287]. Within the brain both isoforms are highly expressed in the cortex and hippocampus. Subcellular localization analysis of hippocampal neurons revealed that p38α is localized in all cellular compartments, including the nucleus, soma, neurites and synapses whereas p38β is predominantly nuclear. Most studies have not distinguished between p38α and p38β in determining their relative contributions to a biological action. During development, p38 MAPK regulates neuronal differentiation, neuronal migration, development of the neuronal skeleton and synapse formation [283,288]. In mature neurons p38 MAPKs regulates ion channel function, axonal transport and axonal regeneration [283]. p38 MAPKs has been shown to regulate learning and memory [289,290]. Several lines of evidence suggest that p38 MAPK, and specifically p38α, regulates synaptic plasticity by promoting synaptic depression and memory [291]. Activation of p38 MAPK contributes to LTP inhibition [292,293,294] and LTD (long-term depression) induction [295,296] in the hippocampus. Interestingly, conditional deletion of p38α in neurons or astrocytes of the mouse hippocampus revealed that deleting neuronal p38α has no effect on LTD whereas deletion in astrocytes abolishes it suggesting that the stimulation of LTD in neurons was driven by its action in astrocytes and establishing a role for p38α in astrocyte-neuron communication. Several studies have described that p38 MAPKs promotes neuronal death induced by a variety of noxious stimuli, including inflammatory cytokines and neurotoxins [297,298], oxidative stress [299,300], hypoxic insult [301], and excitotoxicity [302]. For example, following neuronal damage or injury to the brain or spinal cord, p38 MAPK (and primarily p38α) promotes chronic inflammation through release of cytokines, including IL-1b and TNFa, from astrocytes and microglia, which while designed to be helpful has an inhibitory effect on recovery from injury [181,287,303]. IL-1b and TNFa released by microglia through the action of p38 MAPK also inhibit LTP and therefore synaptic plasticity [304]. Through p38 MAPK, activated astrocytes and glia produce reactive oxygen species that exacerbate the injury to neurons thereby promoting neurodegeneration [181,287]. In addition to causing neuronal dysfunction and death through activation astrocytes and microglia, p38 MAPK can induce death of neurons cell autonomously by activating apoptotic signaling pathways [184,305].

#### 3.2.2. p38 MAPK in AD

p38 MAPK activity is elevated in the hippocampus and cortex in both AD mice and patients relatively early in the disease process [306,307,308,309]. The increase is accompanied by elevated activity of MMK6 on of the two major activators of p38 MAPK [310]. p38 MAPK activation has been observed in both neurons and glial cells. In microglia and astrocytes p38 stimulates chronic release of inflammatory cytokines, which has been shown to be triggered, at least in part, by APP and Aβ that bind to cell surface receptors [181,182,183,198,311,312] (Figure 1B). Although initially helping in the clearance of Aβ, as described above, chronic release of inflammatory cytokines causes neuronal dysfunction and degeneration. A key target of p38 MAPK in its pro-neuroinflammatory role is MK2 (MAPK-activated protein kinase 2). Not surprisingly, MK2 activity is elevated in the brains of AD mice and its genetic deletion in cultured microglia prevents the release of inflammatory cytokines following Aβ treatment. [313]. Within neurons p38 MAPK phosphorylates Tau [187,188], activates pro-apoptotic signaling pathways [182,184,185,186] and promotes excitotoxicity [195,196,197] (Figure 1B). Although all four p38 proteins phosphorylate Tau, pharmacological experiments in mice have identified p38α as the isoform most responsible for Tau phosphorylation at pathogenic residues [189]. Inhibitors selective for p38α suppress neuroinflammation and protect against synaptic dysfunction, cognitive deficits and behavioral deficits in AD mice [189,314]. In these studies the effect of the inhibitors on p38β was not directly assessed, however, and given the structural and functional similarities between the two isoforms, contribution of p38β is possible and perhaps even likely. Some studies have shown that p38 MAPK, and primarily the p38α isoform, inhibits autophagy by phosphorylating and inhibiting the ULK1 complex, a protein complex that initiates the autophagic signaling pathway [199,200,315]. Thus, besides promoting other pathological mechanisms within neurons, p38 MAPK contributes to AD neuropathology by inhibiting autophagy.

Interestingly, and in contrast to p38α (and p38β), p38g has a protective effect in AD mice [316]. This protective effect involves phosphorylation of Tau by p38γ at Ser205 which results in disruption of postsynaptic excitotoxic protein signaling complexes activated by Aβ [316,317]. Neuronal deletion of p38γ in AD mice results in exacerbation of neural circuitry degeneration and cognitive defects as well as premature lethality, demonstrating a protective role for p38γ in AD [316]. Other studies have described that p38γ phosphorylates Tau at Thr50, which enhances the ability of Tau to promote microtubule assembly [187]. In contrast, p38δ phosphorylates Tau at sites that reduce the ability of Tau to promote microtubule assembly [318,319]. Taken together, these results indicate that p38α (and likely p38β and p38δ) promotes AD pathogenesis whereas p38γ exerts a protective role.

#### 3.2.3. p38 MAPK as a Therapeutic Target in AD

Chemical inhibitors selective for p38α have been tested in Aβ and Tau mouse models of AD and found to be effective at reducing inflammatory cytokine production, Tau pathology, synaptic dysfunction and cognitive impairment [320]. Among these are MW01-2-069A-SRM [314,320], MW181 [189] and MW150 [192,321], and VX-745 [322]. Administration of NJK14047, a p38 MAPK inhibitory compound with demonstrated anti-inflammatory effects, was found to reduce microglial-induced neuroinflammation as well as Aβ deposition, neurodegeneration and memory impairment in the 5XFAD mouse model of AD [323]. As described above, besides promoting neurodegeneration by stimulating chronic inflammation, p38 MAP directly causes neuronal death by activating pro-apoptotic pathways and increasing levels of reactive oxygen species [182,184,186]. A study conducted in rats showed that the p38 MAPK inhibitor, PD169316, blocked pro-apoptotic signaling and reduced neuronal loss induced by intracerebroventricular injection of Aβ [324]. p38 MAPK inhibitors have been shown to attenuate Aβ-induced LTD impairment in hippocampal and entorhinal cortex slices [192,193,194]. Protection against cognitive impairment in AD was described with another p38 MAPK inhibitor possessing anti-inflammatory effect [325]. Another study described reduction of neuroinflammation and cognitive impairment through administration of a peptidic MK2 inhibitor, MMI-0100 [326]. As described above, MK2 is a downstream target of p38 MAPK.

Other p38 MAPK inhibitors have been reported to have beneficial effects in cell culture and rodent models of AD but the selectivity of these inhibitors for p38 MAPK has not been well-documented [327,328,329,330,331]. Some studies have used p38 MAPK inhibitors that are selective, but their ability to suppress neuropathology or improve behavioral performance *in vivo* was not been adequately evaluated [332].

#### 3.2.4. p38 in HD

p38 MAPK activity is increased in the stratum of HD patients and in mouse models and this increase has been found to be associated with neuronal death [195,333,334,335]. One study attributed the increase in p38 MAPK activity to reduced activity of MKP-1, a phosphatase that inactivates p38 MAPK by dephosphorylation of the Thr-Gly-Tyr motif [336]. Pharmacological stimulation of MKP-1 reduces p38 MAPK activity and protects against the neurotoxic effect of mut-Htt expression in cultured cells and in mice with striatally-injected mut-Htt [336]. Neurodegeneration caused by mut-Htt is, at least in part, due to NMDA receptor-mediated excitotoxicity. Excitotoxic death in HD has been found to involve enhanced interaction between the NMDA receptor and the postsynaptic protein PSD95, which through abnormal activation of calpains results in the cleavage of STEP61 a phosphatase enriched in the striatum [337]. Whereas full-length STEP61 negatively regulates p38 MAPK in striatal neurons, calpain-cleaved STEP61 cannot dephosphorylate p38 MAPK and therefore unable to suppress excitotoxicity [337]. Calcineurin, is yet another phosphatase, that enhances mut-Htt neurotoxicity both in cell culture and in the striatum of HD mice by dephosphorylating at Ser421 of mut-Htt [338]. Pharmacological inhibition of calcineurin with FK506 results in elevated Ser421 phosphorylation, which protects against mut-Htt neurotoxicity [263,338,339]. SGK (serum-and glucocorticoid induced kinase) is a kinase that phosphorylates mut-Htt at Ser421. Somewhat counterintuitively, increased p38 MAPK activity in HD leads to induction of SGK activity in the striatum and cortex [334]. It is likely that the induction of SGK following p38 activation reflects a stress response in neurons degenerating due to p38 MAPK activation. p38 MAPK suppresses chymotrypsin-like protease activity leading to the accumulation and aggregation of mut-Htt [340]. Pharmacological inhibition of p38 MAPK leads to an increase in chymotrypsin-like protease activity and consequently enhanced clearance of mut-Htt.

#### 3.2.5. p38 MAPK as a Therapeutic Target in HD

Studies using HD mice have found that the increased activation of p38 MAPK correlates with striatal degeneration indicating a causal role for p38 MAPK in HD-associated neurodegeneration [333,337]. The p38 MAPK inhibitor, SB-239063, protects striatal neurons cultured from HD mice from degeneration [195]. Similar neuroprotective effects of another p38 MAPK inhibitor, SB203580, was described both in HD mice and striatal cells overexpressing mut-Htt [341]. The mechanism by which p38 MAPK inhibition protects in mouse HD models is not fully clear but likely involves blockade of apoptotic signaling within neurons as well as inhibition of glia-mediated neuroinflammation. Other mechanisms by which p38 MAPK inhibitors could protect is through inhibition of calcineurin and activation of chymotrypsin-like protease activity, as described above.

### 3.3. (C) Cyclin-Dependent Kinases

The eucaryotic cell cycle is comprised of four phases – G1, S, G2 (phases during which the cell grows and duplicates its DNA) and M phase when mitosis occurs. Quiescent cells that lack sufficient nutrients to traverse the cell cycle reside in the G0 phase, a phase in which neurons and other postmitotic cells also reside. Two check-points, G1/S and G2/M, ensure that the cell is ready for DNA replication and that DNA replication is accurately completed, respectively. Progression through the various phases of the cell cycle is driven in large part by cyclin-dependent kinases (CDKs), a family of serine-threonine kinases. Activation of CDKs require heteromerization with specific cyclins, such that each CDK has one (or sometimes two) cognate cyclin protein interacting with and activating it [342,343,344,345]. Cyclin binding, which involves a motif called the cyclin box, changes the conformation of the CDKs to expose the substrate-binding region of the kinase [345,346]. The levels of most cyclins oscillate within the cell such that they are synthesized as the cell cycle enters the phase in which the cyclin is needed and rapidly degraded on exiting it which inactivates its CDK. Whereas many of the approximately 21 CDKs expressed in mammals play various roles including the regulation of transcription, cell cycle progression is under the charge of a core group of CDKs, including CDK1, 2, 3, 4 and 6.

The transition from G0 to G1 requires the activity of CDK3/cyclin C whereas CDK4-cyclin D and CDK6-cyclin D promote passage through G1. A key substrate of both CDK4 and CDK6 is the retinoblastoma protein (Rb) which sequesters the transcription factor E2F1 by interacting with it. Hyperphosphorylation of Rb by CDK4 and CDK6 during the G1 phase causes its disassociation from E2F, allowing E2F1 to bind to the promoters and induce expression of cell cycle promoting genes, including the cyclin A and E genes. The CDK2-cyclin E/A complexes promotes transition through S-phase followed by CDK1/Cyclin A- and CDK1/Cyclin B-mediated passage through G2, M-phase and cytokinesis. The activity of the CDK-cyclin complexes are inhibited by CDK-inhibitory proteins (CKIs) which act at different phases to slow down or prevent cell cycle progression. A multitude of other protein, including transcription factors and various inhibitory and activating kinases and phosphatases, modulate the activity of CDK-cyclins on cell cycle progression [343,344].

Neurons are kept in G0 through the constant inhibition of cell cycle-promoting CDKs along with activation of CDK inhibitors. However, aberrant activation of CDKs, often resulting from the loss of inhibitory mechanisms, allows neurons to enter the cell cycle leading to their death by apoptosis [34,347,348,349]. Increasing E2F1 levels by ectopic expression (which is normally caused by CDK4 and CDK6) induces apoptosis in neurons whereas suppression of E2F1 expression is neuroprotective in cultured neurons exposed to a variety of death-inducing stimuli and in animal models of neurodegeneration [350,351,352,353,354,355]. Interestingly, E2F1 is phosphorylated both in culture and *in vivo* by GSK3 and p38 MAPK [178,190,191]. Indeed, phosphorylation by GSK3 is necessary for E2F1-induced neuronal death [179]. In addition to E2F1, pharmacological inhibition of GSK3 also inhibits of expression of cyclin D, cyclin E, Rb phosphorylation and the activity of E2F1 [356]. These results indicate cross-talk between the cell cycle machinery and signaling by GSK3 and p38 MAPK.

An unconventional and somewhat unique member of the CDK family is CDK5, a widely-expressed protein that is not activated by cyclins and does not directly participate in cell cycle progression. CDK5 is activated through interaction with either of two activating proteins, p35 and p39, that are expressed only in the nervous system and predominantly in postmitotic neurons [357]. Because of the restricted expression pattern of its activating proteins, the kinase activity of CDK5 is restricted to the CNS where it is involved in a variety of critical functions, particularly during brain development but also during adulthood [357].

### 3.4. Role of CDKs in the Brain

Development of the mammalian brain requires rapid proliferation of neuroepithelial cells and their derivatives, neural progenitor cells (NPCs), which results in increased brain mass [358,359,360]. After a period of proliferation, increasing numbers of NPCs exit the cell cycle and start differentiating into neurons and other brain cell types [358,359,360]. Careful regulation of CDKs and other cell cycle components is critical for proper development and functioning of the brain. Deregulation of CDKs, their cyclin partners, or their regulators affects not only the generation of the proper number of NPCs but also the timing and extent of their differentiation to generate the proper number of postmitotic neurons and glial cells. Indeed, disruption of the balance between NPC production and cell cycle exit impacts neuronal differentiation and migration, and underlies a variety of neurodevelopmental disorders [361,362,363]. While primarily involved in promoting NPC proliferation, the presence of CDKs 1, 2 and 4 and their cognate cyclins are detectable in dendrites and axons where they inhibit neuronal maturation [364,365,366]. Abnormal increases in the activity of these CDKs result in aberrant phosphorylation of proteins in axons and dendrites, including Tau [367]. In contrast to the cell cycle-promoting CDKs, CDK5 promotes differentiation, maturation and migration of postmitotic neurons on the developing brain [360]. In the adult brain CDK5 plays a key role in synaptic plasticity, learning and memory [368,369]. CDK5 also prevents neurons from entering the cell cycle through interaction with E2F1. However, and as described below, overactivation of CDK5 can negatively brain function and can promote neurodegeneration [370,371,372]. One study has described that normally CDK5 activity is prevented from becoming overactive by cyclin E through direct binding [373]. Genetic ablation of cyclin E, which is expressed at high levels in terminally differentiated neurons, results in abnormal activation of CDK5 reduced number and volume of dendritic spines and impaired synaptic plasticity and memory [373].

#### 3.4.1. CDKs in AD

A compelling body of evident implicate aberrant CDK activation and cell cycle entry as being causally involved in AD pathogenesis. Death of cultured cortical and hippocampal neurons resulting from Aβ treatment is preceded by induction of various cyclins and activation of CDKs and can be prevented by treatment with broad spectrum CDK inhibitors [374,375,376,377]. Increased activity of specific CDKs and/or cyclins or deregulation of other cell cycle-modulating proteins has also been described in vulnerable regions of the brain in both AD mice and patients [34,35,36,201,202,203]. In cycling cells expressing Tau, cell cycle progression coincides with Tau hyperphosphorylation and altered microtubule stability [378,379]. Similarly, in the AD brain activation of the cell cycle is believed to contribute to Tau hyperphosphorylation and aggregation as well as microtubule destabilization [201,380]. However, one study described that Aβ-induced stimulation of cell cycle markers requires Tau as the increase is not seen in Tau-deficient neurons [381]. Taken together, these findings suggest the possibility of a feed-forward loop in which deregulated cell cycle components cause Tau phosphorylation, which in turn stimulates the cell cycle. Intracerebral infusion of Aβ in mice results in increased expression of mitotic proteins prior to memory deficits, both of which are prevented by co-administration of the CDK inhibitor, flavopiridol, suggesting CDKs involvement in AD-related memory impairment [45]. Although activation of cell cycle components by Aβ has been well-documented, some studies have concluded that cell cycle deregulation occurs early in the disease process, prior to the onset of neuropathology [201,382]. Indeed, cell cycle-promoting CDKs phosphorylate APP while their inhibition reduces Aβ production resulting in decreased synapse loss and memory impairment in AD mice [383]. Microglia activated by Aβ can induce cell cycle deregulation and death of co-cultured neurons demonstrating that glial signals contribute to the neurotoxic effects of Aβ by activating CDKs [384]. CDK inhibitors inhibit neuropathological abnormalities and reduce behavioral deficits in AD mice in several studies [40,45,202]. One analysis of the literature reported that 13 of 37 AD-risk genes are likely to be functionally involved in cell cycle or mitosis regulation [36]. In sum and with regards to where in the cell cycle neurons die, there is consensus from cell culture and mouse models (as well as some data from postmortem samples from AD patients) that in the AD brain neurons leave G0 to enter the cell cycle but are unable to traverse through S-phase and complete mitosis resulting in their death by apoptosis [34,35,36,201,385,386]. Some evidence indicates that neurons are arrested at cell cycle checkpoints, most commonly at G2/M, prior to dying by apoptosis, [387,388]. While most evidence points to defects prior to the M-phase in AD models, upregulation of mitotic CDK activators and downregulation of mitotic CDK inhibitors has been observed as well [389,390].

Although aberrant cell cycle entry is the most described effect of activated cell cycle-related CDKs in neurons, other mechanisms by which these CDKs promote neuropathology have been described. For example, activation of CDK1 increases APP processing to Aβ and impairs autophagy in AD through inhibition of Beclin-1 [204]. CDK1 can phosphorylate Aβ at Ser26 increasing its neurotoxicity and reducing its ability to form insoluble fibrils [391,392]. CDK1 and CDK2 also contribute to Tau hyperphosphorylation [201,393,394,395,396]. In cell culture experiments, toxicity by Aβ was shown to involve induction of CDK2 activity and its phosphorylation of Tau [397] (Figure 1C)

The CDK believed to be most involved in AD pathogenesis is CDK5. Proteolysis of p35 (and p39) by an increase in calpain activity produces a fragment, p25 (and p29), that forms a highly stable complex with CDK5 causing its hyperactivation [398]. Because p25 is cytosolic, CDK5 activity is also shifted from the membrane, where p35 is localized, to the cytosol. The mislocalization of CDK5 to the cytoplasm lies results in the phosphorylation of proteins that are not natural substrates of the kinase. As described below, one of these CDK-phosphorylated cytosolic protein is Tau. Indeed, in addition to GSK3, CDK5 is considered to be another major Tau kinase (Figure 1C). Hyperactive CDK5/p25 phosphorylates Tau at sites that are phosphorylated in the brains of AD patients and promotes its dysfunction [209]. Transgenic mice in which p25 is expressed in the forebrain inducible display neurodegeneration, neuroinflammation and cognitive deficits [399,400,401]. Hyperactive CDK5 also increases production of Aβ by both transcriptional mechanisms [205] and by stimulating the activity of BACE1 [206] and PS1 [207] (Figure 1C). An early consequence of the CDK5-mediated increase in Aβ is an impairment of synaptic homeostasis [205]. Increased Aβ can also activate CDK5 and increase Tau hyperphosphorylation suggesting a positive feedback loop [204,402]. Enhanced CDK5 activity in glial cells promotes neuroinflammation by increasing production of lysophosphatidylcholine, which induces release of inflammatory cytokines [212]. Other studies have found that CDK5 stimulated by Aβ exposure activates CDKs1, 2 and 4 by phosphorylating them along with inactivation of phosphatases that negatively regulate these CDKs [377]. Once activated the CDKs phosphorylate lamins (which normally occurs during mitosis in dividing cells) resulting in damage of the nuclear envelope by phosphorylating [208]. It is widely recognized that in AD (and other neurodegenerative diseases) the nuclear membrane is deformed and nucleocytoplasmic transport is dysfunctional [403,404]. It deserves mention that the results of studies by some have disputed the aforementioned p25-mediated CDK5 hyperactivation model initially proposed by Patrick, Cruz and Tsai et al. [369,405]. These laboratories propose that p25 is produced normally, has an important role in memory and learning, and that its expression is actually reduced in the AD brain contributing to cognitive deficits [369,405]. Regardless of whether activation results from p25 production or through other mechanisms, it is widely accepted that CDK5 activity is elevated in the AD brain and is a key contributor of disease pathogenesis [406,407,408]. Interestingly, recent studies have described that like GSK3 and p38 MAPK, CDK5 also inhibits autophagy although acting through a different mechanism involving phosphorylation of the Vps34 protein which interferes with its interaction with Beclin-1, an interaction required for the initiation of autophagy. Besides affecting clearance of pathogenic Aβ and Tau, one study has shown that CDK-mediated inhibition of autophagy deregulates APP processing [204,210,211].

#### 3.4.2. CDKs as a Therapeutic Target in AD

Many studies have described protection by CDK inhibitors against Aβ-induced toxicity in cultured neurons [34,35,36,201,385,386]. CDK inhibitors have also been successfully tested in AD mice. In one mouse study administration of the non-selective CDK inhibitor flavopiridol in mice prevented the increased expression of cell cycle proteins and reduced memory impairment resulting from intrecerebroventricular injection of oligomeric Aβ [45]. Similar findings were described in another study in which administration of another non-selective CDK inhibitor, roscovitine, reversed disease-associated transcriptomic changes, reduced Aβ and Tau pathology, and improved behavioral performance in an AD mouse model [202]. Most emphasis on CDKs as a therapeutic target has been on CDK5. Intracerebroventricular infusions of the CDK5 inhibitory peptide (CIP), a 125 aa peptide generated from p35 by C- and N-terminal cleavage inhibits CDK5/p25 activity and reduces Tau hyperphosphorylation, the number of NFTs and neurodegeneration in the p25-overpressing transgenic mouse model of AD [409]. Other studies in which CIP was delivered through an adeno-associated viral vector was similarly protective in p25-transgenic mice reducing neuroinflammation, neuropathology and neurodegeneration [410,411,412]. Administration of another 20 aa peptide derived from p35 cleavage, TFP5, and also inhibits CDK5/p25 activity, reduces neuropathology and restores synaptic function and cognitive function in both the 5XFAD and p25-overexpressing mice. Although derived from p35 TFP5 does not inhibit endogenous CDK5/p35 activity or the activity of other cell cycle-promoting CDKs [413,414]. Interestingly, and in contrast to CIP, TFP5 was shown to be brain permeable increasing its potential as a therapeutic agent. Pharmacological inhibition of CDK5 prevents the reduction of hippocampal neurogenesis in adult AD mice although the inhibitor used in the study, roscovitine, also inhibits cell cycle-promoting CDKs [415,416]. Another study reported that the anti-diabetes drug pioglitazone, a thiazolidinedione compound, inhibits CDK5 activity by decreasing p35 protein level [417]. Pioglitazone restored LTP in Aβ-treated hippocampal slices and reduced memory deficits in AD mouse models [417]. However, pioglitazone has many effects including regulating insulin signaling and PPARg [418]. Despite these limitations, inhibition of CDK5 activity represents a promising starting point in the development of treatments for AD.

#### 3.4.3. CDKs in HD

Multiple lines of evidence supports a role for deregulated expression of cell cycle components and inappropriate entry of neurons in the cell cycle in the pathogenesis of HD. Expression of mut-Htt is sufficient to cause cell cycle defects in tissue culture models leading to cell death [419,420,421,422]. HD mice and cultured neurons expressing mut-Htt displayed induction of cyclin B activity [422]. In HD mice, reactivation of cell cycle markers was found in early and middle stages of the disease process. Increased expression of E2F1, cyclin D1and cyclin E, all necessary for progression through the G1 phase, has been found in the striatum of HD striatum and HD mice [419,420]. Another study described increased phosphorylation of Rb and reduced expression of the CDK-inhibitory protein, p27, in HD mice and in a cell culture model of HD [423]. Treatment with CDK inhibitors was protective in the cell culture HD model. Using a mouse model and careful examination of the striatum one study reported that cell cycle entry was preceded by perinuclear accumulation of mut-Htt and damage of the nuclear membrane [422]. The induction of cell cycle entry and neuronal death by perinuclear accumulation of mut-Htt was confirmed in cultured cortical neurons [422]. Deregulation of the ER stress response pathway resulting from reduced ATF6α/Rheb (Ras-homologue enriched in brain) signaling is another mechanism that has been proposed to induce cell cycle reactivation and death of neurons [420]. A network analysis of human post-mortem microarrays identified CDK1 as one of 19 genes that were particularly significant to HD pathogenesis [424]. Although most of the cell culture and mouse experiments describe induction of cell cycle proteins operating at G1 and S phases, this finding suggests that deregulation of M-phase CDKs are also important. In sum, although the evidence for cell cycle reactivation in HD is strong, the roles of individual CDKs and cyclins in causing neuronal death is unresolved.

Compelling evidence links deregulated CDK5 activity to HD pathogenesis. CDK5 activity is elevated in HD mice and patients [425] and in mice, contributes to behavioral abnormalities characterizing the disease and neurodegeneration. In HD patients, cognitive disturbances and learning and memory deficits manifest well before motor dysfunction [426,427,428]. In HD mice, the genetic knockdown of CDK5 expression attenuates these progressive cognitive and memory impairments [429]. This recovery was attributed to readjusting levels of specific glutamate receptor subunits and restoring hippocampal spine density [429]. Depressive behavior has been suggested to correlate with cognitive disturbances in HD and reflects severity of impaired cognitive performance [430]. Deregulation of CDK5 activity contributes to depressive-like behavior in HD mice acting through aberrant phosphorylation of DARPP-32 (dopamine- and cAMP-regulated phosphoprotein 32), a protein expressed selectively in the striatum [216]. This suggests that CDK5 mis-regulation could affect multiple HD-associated symptoms. Both genetic knockdown and pharmacological inhibition of CDK5 with roscovitine ameliorates depressive-like behavior in HD mice [216]. A defining feature of HD is involuntary motor movements, which results from degeneration of GABAergic neurons in the striatum. Different laboratories have concluded that elevated CDK5 activity contributes to striatal neurodegeneration. One study described increased production of p25 and elevated CDK5 phosphorylation induces oxidative stress and NMDA receptor activity in striatal neurons increasing their vulnerability to death [214,215]. Oxidative stress and NMDA receptor activation are known to cause excitotoxicity which is considered to be the major mode of neuronal death in HD [65,431]. One study found that CDK5/p25 promotes excitotoxicity by phosphorylation and destabilization of Fbxw7 (F-box/WD repeat-containing protein 7), which results in increased expression of the pro-apoptotic protein, c-jun [217]. Another study described that in response to NMDA receptor stimulation CDK5/p25 phosphorylated and destabilized Cdh1 causing the stabilization and accumulation of cyclin B1 and leading to apoptotic death [218]. Genetic reduction of p25 or p35 in HD mice attenuated CDK5 hyperactivity and protects against NMDA receptor-mediated excitotoxicity [432]. Striatal neurons are sensitive to dopaminergic toxicity [77,433]. CDK5 has been found to increased dopamine neurotoxicity in HD models an action involving increased mitochondrial fission [213]. Pharmacological inhibition of CDK5 reduces mitochondrial fission and protects against dopamine toxicity [213].

It is noteworthy that although the overwhelming consensus is that CDK5 activity is elevated in HD and contributes to disease pathogenesis, two separate studies have described that CDK5 phosphorylates mut-Htt and that this modification protects it from cleavage to the toxic N-terminus fragment [434]. In one of these studies it was found that CDK5 activity was reduced by expression of mut-Htt through direct binding which prevented the interaction of p35 with CDK5. The reduction of CDK5 activity enhanced cleavage of mut-Htt and therefore increased neurotoxicity in neuronal cell lines [434]. Protective phosphorylation of mut-Htt by CDK5 was confirmed in another study in primary striatal neurons from HD mice and subjected to DNA-damage using camptothecin [435]. This study also described that even wild-type Htt (which is also phosphorylated by CDK5) is rendered neurotoxic in response to DNA-damage if CDK5-mediated phosphorylation is inhibited [435]. These authors described a substantial reduction of both CDK5 and p35 levels in the striatum of patients with late-stage HD. The expression of p25 was not studied in the two studies and the issue of whether mut-Htt disrupts CDK binding to p25 is unclear. Therefore, while the overall consensus supports a role for a causal role for elevated CDK5 in HD, more research is necessary to establish this unequivocally.

#### 3.4.4. CDKs Are a Therapeutic Target for HD

Chemical inhibitors of CDK1 and CDK2 protect against neurodegeneration in the 3-NP (3-nitropropionic acid)-induced mouse model of HD [436]. Genetic knockdown of CDK5 protects against corticostriatal learning deficits, hippocampal-dependent memory impairment and depressive behavior in HD mice [216,429]. As indicated above, CDK inhibition provides protection against dopaminergic toxicity and aberrant mitochondrial fission in a cell culture model of HD [213], both that are believed to play key roles in disease pathogenesis [65,437,438]. Results of some studies suggest that whereas CDK5/p25 promotes HD neuropathology, CDK5/p35 may have protective effects. Consistent with this, knockdown or inhibition of CDK5/p35 has been found to have either no effect or a negative effect in HD cells and mice [434,439,440]. Although yet to be rigorously tested and confirmed, this raises the possibility that CDK5-based HD therapeutics will have to selectively target CDK5/p25.

### 3.5. Would Inhibiting Any One Target Work for AD and HD? The Case for Multi-Target Therapies

Intense effort has been made over the past two decades to develop an effective disease-modifying therapy for AD. Much of this effort has targeted Aβ and Tau. These include active and passive immunotherapy against Aβ and Tau, inhibitors of their oligomerization and fibrillization, inhibitors of Aβ production and enhancers of its clearance, and inhibitors of Tau-phosphorylating kinases and microtubule stabilizers [57]. However, it is now well-accepted that the pathogenic mechanisms underlying AD are complex and involve critical contributions from multiple neuronal and glial cell types and molecular targets within and outside them in addition to Aβ and Tau. Even within vulnerable regions of the AD brain there can be diverse signaling mechanisms that contribute to disease pathogenesis. For example, results from single cell transcriptomic analysis indicate that AD pathology-related gene expression changes can be both cell-specific as well as common across cell types in the brain [441]. The cell-specific changes were found to be diverse. Moreover, the transcriptional profiles were different between sexes in several cell types [441]. A more recent single-nucleus transcriptomic study conducted both tissue from 5XFAD mice and AD patients with TREM2 mutations found that transcriptional signatures in human AD in microglia, astrocytes and oligodendrocytes were strikingly different from those observed in mice [442]. Surprisingly, there was limited concordance between the two aforementioned transcriptomics studies [441,442]. To add to the complexity, recent neuropathological studies indicate multiple subtypes of AD with distinct clinical presentation, age at onset, disease duration, and rate of cognitive decline [443]. A study that utilized PET scanning to study spatiotemporal spreading of Tau in living patients described four distinct spreading and deposition patterns of Tau that presented with distinct cognitive profiles and disease progression patterns [444]. Along with the recognition that the disease process in AD begins many years before Aβ and Tau pathology is detectable suggests that therapies should simultaneously target multiple disease-relevant molecules that act upstream of Aβ and Tau abnormalities.

As described in this review, kinases including GSK3, p38 MAPK and CDKs affect disease initiation and progression in multiple ways acting both upstream and during neuropathology and cognitive impairment. For example, in the case of p38 MAPK, contributions to AD are cell autonomous (stimulating cell death mechanisms within neurons) and non-cell autonomous (through release of cytokines from astrocytes and microglia). A recent study described Tau pathology in oligodendrocytes in AD mice where it co-localized with active p38 MAPK, which regulated Tau seeding [445]. An attractive therapeutic approach would be to identify drugs that target multiple molecules or chemically modify existing single-target drugs to regulate additional targets. Some such drugs, initially believed to be selective again a target, have subsequently been found to inhibit other targets relevant to disease pathology. Examples of such drugs described in this review include lithium, valproic acid, flavopiridol, roscovitine and rapamycin. Kenpaullone, a compound developed and often used as a CDK inhibitor, also inhibits GSK3 and has been utilized in some studies as a GSK3 inhibitor [446,447]. Kenpaullone reduces phosphorylation of APP and lowers its processing to Aβ [447]. Recent efforts in the development of novel treatments for human diseases has led to the synthesis of multi-kinase inhibitors [448]. Rational designing has generated other compounds that inhibit CDK1/GSK3 [449,450], CDK1/CDK5/GSK3 [451,452,453], CDK1/CDK2/CDK5/GSK3 [454] and p38 MAPK/GSK3 [455]. Recently dual CDK5/GSK3 inhibitors possessing a tetrahydropyridine isoindolone skeleton have been identified [456,457]. Using a different approach another CDK5/GSK3 inhibiting compound, LDC8, was identified and shown to protect against neuroinflammation-induced neuronal death in vitro [458]. In comparison, genetic knockdown of CDK5 displayed only partial protection indicating that simultaneous inhibition of both GSK3 and CDK5 is necessary for complete protection. A few of these multi-kinase inhibitors have been tested in model systems as candidates for AD therapeutics. LDC8 was also found to protect against neuroinflammation and synaptic degeneration in a zebrafish model of AD [458]. Screening of benzofuropyridine compounds for their ability to inhibit phosphorylation and oligomerization of Tau has led to the identification of a compound that inhibits GSK3β, CDK1 and CDK5 [396]. The triple-kinase inhibitor was more effective at inhibiting Tau phosphorylation and oligomerization in cultured cells than other compounds identified in the screen that inhibited only one or two of the kinases [396]. Another study reported that dihydroxy-1-aza-9-oxafluorene compounds that inhibit CDK1/GSK-3β/CDK5/p25 robustly inhibit Tau phosphorylation at nanomolar concentrations [452]. Interestingly, a compound, HSB13, which inhibits all the AD-causing kinases covered in this review - GSK3, CDKs1,2 and 5 and p38 MAPK - has been shown to protect against neurodegeneration in a *Drosophila* model of AD [459]. The potential of multi-kinase inhibitors that target kinases discussed in this review is further elevated by the finding of cross-talk between their signaling actions in the disease process. For example, elevated CDK5 has been proposed to cause neuronal damage and cognitive impairment in mice by stimulating GSK3 signaling [460]. Another study described that GSK3 binds and is activated by p25 [461]. Surprisingly, while binding p25 more effectively than CDK5, GSK3 does not bind to p35. Finally, the action of pharmacological inhibitors, including kinase inhibitors, are easier to titrate than knockdown approaches or immunotherapy. Since most of the molecules that are currently being targeted, including Aβ and Tau and enzymes regulating their production, have important physiological functions, partial inhibition of the targets, which would normalize the pathological increase in activity without completely neutralizing it, could be better than potent inhibition in reducing unwanted actions of treatment. For example, GSK inhibition required for neurological benefit is much lower (20–25% inhibition) than what is required for stabilization of β-catenin, a major cellular target of GSK3 [462,463]. In this regard it is interesting that while inhibiting GSK3, p38 MAPK and CDKS, HSB13, does so partially suggesting that drugs like it might be good candidates for clinical and pre-clinical testing [459].

Although a monogenic disease, HD pathogenesis is also a highly complex process involving many molecules and signaling pathways. Again, targeting multiple disease-relevant molecules simultaneously could represent a more effective approach than the dominant current strategy of reducing mut-Htt levels through antisense technology. Support for this comes from studies in HD mice in which inhibitors of two or more signaling molecules was found to be more effective than inhibition of a single target. For example, in a study using two types of HD mice, benefit on motor and cognitive impairment was observed only when lithium (which inhibits GSK3) and valproic acid (which inhibits HDACs) were co-administered. A study conducted in an HD fly model described that co-treatment with rapamycin (an mTOR inhibitor) and lithium displayed significantly more protection that with either inhibitor alone. The multi-kinase inhibitor, HSB13, displays strong efficacy against neurodegeneration and behavioral deficits in the 3-NP model of HD [459].

## 4. Future Directions

With the recent advances in in silico drug design (or computer-aided drug design) for neurodegenerative diseases it would be possible to more rapidly design drugs that effectively inhibit two or all three of the key kinases covered in this review [464,465,466]. Development in high-throughput screening platforms that utilize cultured cells or invertebrate models to test large numbers of candidate drugs for multi-kinase inhibition as well as neuroprotection, cytotoxicity and Aβ/Tau/mut-Htt aggregation is also facilitating the development of novel pharmacotherapeutic. Strategies [467,468,469]. Advances in the development of novel preclinical platforms, such as patient-derived iPSC (induced pluripotent stem cells) and 3D organoids, are bridging the translational gap between animal models and human clinical trials [470,471]. An issue that cannot be ignored with regard to therapeutics for neurodegenerative diseases, such as AD and HD, is the delivery of drugs to vulnerable brain parts given the presence of the blood-brain barrier and the large number of efflux transporters in the CNS. Considerable effort has been placed in recent years on the development of nanoparticles as drug delivery vehicles [472,473,474]. A number of nanoparticles, including metal nanoparticles, solid lipid nanoparticles, polymeric nanoparticles, liposomes and extracellular vesicles (EVs) have been used in experimental models of AD and neurodegenerative disease [472,473,474]. Of these polymeric nanoparticles (including hydrogels), liposomes and EVs appear to be particularly attractive because they are more biocompatible and biodegradable. In the case of the three kinases described in this review, nanoparticles can be loaded and encapsulated with chemical inhibitors that reduce enzyme activity, but also biologicals to knockdown their expression including microRNAS (mRNAs) and antisense oligonucleotides [475]. An attractive approach for use in brain pathologies is nasal delivery of lipid nanoparticles and drugs, which has been used in patients for many disorders and ailments, but is now being actively developed for AD and other neurodegenerative diseases [476,477].
